# Metabolomics and electronic tongue analysis show taste differences in Juhong tea from various black tea sources

**DOI:** 10.1016/j.fochx.2026.104618

**Published:** 2026-10-12

**Authors:** Anhui Gui, Fei Ye, Lin Feng, Shengpeng Wang, Panpan Liu, Jinbo Liang, Xueping Wang, Jinjin Xue, Jun Xiang, Fangjun Su, Jing Teng, Jing Huang, Pengcheng Zheng, Shiwei Gao

**Affiliations:** a Key Laboratory of Tea Resources Comprehensive Utilization, Ministry of Agriculture and Rural Affairs, Hubei Tea Engineering and Technology Research Centre, Fruit and Tea Research Institute, Hubei Academy of Agricultural Sciences, Wuhan, Hubei, 430064, China; b Enshi Tujia and Miao Autonomous Prefecture Academy of Agricultural Sciences, Enshi 445000, Hubei, China; c Hubei Jinguo Tea Industry Co., Ltd. in Enshi Tujia and Miao Autonomous Prefecture, Enshi 445000, China

**Keywords:** Juhong tea, Taste profile, Electronic-tongue, Antioxidant activity

## Abstract

Juhong tea is an innovative blend of citrus peel and black tea characterized by a multifaceted flavor system and synergistically enhanced bioactive constituents. Here, the taste profiles, metabolic variations and antioxidant ability were comprehensively compared and characterized across four Juhong teas derived from diverse raw materials: traditional Juhong tea (TJH), single-bud Juhong tea (BJH), wild Juhong tea (WJH), and shaded Juhong tea (SJH), with both commonalities and distinctions highlighted. Sensory evaluation showed that BJH received the highest average overall sensory score (8.05), driven by a rich profile of amino acids (totaling 20.56 mg/g), whereas TJH exhibited superior sweetness (8.85) in electronic tongue analysis due to higher soluble sugar and theaflavin concentrations. Widely targeted metabolomics identified 520 nonvolatile metabolites across the samples, with 193 key differential metabolites screened via OPLS-DA by the criteria of variable importance in projection (VIP) > 1, *p* < 0.05, and |log2(fold change) | > 1. The common abundant metabolites, particularly theanine, 2-aminoisobutyrate, tangeretin, and hesperidin, synergistically shape the characteristic umami, sweet, and citrus-like flavor of Juhong teas. Furthermore, correlation analysis indicated that polyphenols, amino acids, and theaflavins jointly drive the antioxidant properties of Juhong tea. Collectively, these findings revealed the chemical basis of the Juhong tea flavor, providing a theoretical framework for quality control and processing optimization of citrus-tea composites.

## Highlights

• Four Juhong teas from different black tea bases showed distinct taste profiles.
• Widely targeted metabolomics revealed 193 key differential nonvolatile metabolites.
• Theanine, hesperidin, and theaflavins were linked to the flavor and antioxidant activities of Juhong teas.

## 1 Introduction

Citrus tea, a reprocessed composite tea, has recently gained popularity in the Chinese tea market. The earliest typical representatives of this type of tea were Ganpu tea from Yunnan, Guangdong, and Fujian province (Xu et al., 2021). Traditional preparation usually involved scooping out the fresh citrus fruit pulp and filling it with Pu’er tea, followed by sun-withering, temperature-controlled baking, or direct drying (X. Chen et al., 2017; X. Deng et al., 2023; Zhou, Zhang, Liu, Zhang, Wu, & Wu, 2023). The increasing consumer demand for diversified flavors and health functions, shifted research and development from Pu’er tea to a broader range of tea combinations. Among them, Juhong tea, an innovative blend of citrus peel and black tea, is becoming increasingly relevant. This combination not only inherits the sweet and mellow characteristics of black tea but also endows the product with active components from the citrus peel. However, compared with citrus Pu’er tea, Juhong tea differs in the tea base, citrus varieties, and flavor chemistry. Currently, systematic research on Juhong tea is still lacking.

Tea is abundant in diverse secondary metabolites, including tea polyphenols, alkaloids, free amino acids, phenolic acids, organic acids, and tannins, that are intricately linked to tea quality. These bioactive compounds collectively shape the complex taste profile and distinctive flavor characteristics that define premium tea products (Farag et al., 2023). Meanwhile, citrus peels serve as an abundant reservoir of bioactive constituents, particularly flavonoids, phenolic acids, and their derivatives. These compounds exhibit a broad spectrum of therapeutic potentials, encompassing antioxidant, anti-carcinogenic, anti-inflammatory, and anti-atherosclerotic activities (X. Chen et al., 2017; Revathi et al., 2025; Zheng et al., 2024). Juhong tea is produced by packing black tea into fresh citrus peels, followed by a roasting process. This unique manufacturing approach integrates the metabolic attributes of both the tea leaves and the citrus rind. While the theaflavins, thearubigins, and polyphenol derivatives generated during black tea fermentation provide antioxidant and digestive benefits (Babich et al., 2008; Shang et al., 2021; Shi et al., 2024), the co-processing with flavonoid-rich citrus peels creates a more complex flavor profile and enhances the product's bioactive content (Zhou et al., 2023). Specifically, monoterpenoid volatiles like limonene significantly bolster the infusion's fruity fullness(D. Wang et al., 2026). Simultaneously, flavonoids such as hesperidin work synergistically with free amino acids from the tea to temper inherent bitterness and astringency, resulting in a notably more mellow and balanced palate (Jiang et al., 2019; Kaneko et al., 2006; Scharbert et al., 2004).

Current research focuses on changes in bioactive components and flavor characteristics using drying and processing of citrus tea products (Yan et al., 2023; Zhou, Zhang, Liu, Zhang, & Wu, 2023). Significant variations in volatile oils and flavonoids among different citrus varieties directly dictate the characteristic aroma and taste of the final product (Guo et al., 2025; J. Wang et al., 2020). Nevertheless, how diverse black tea bases influence the flavor characteristics of Juhong tea remains unclear. Fresh leaves from different grades and cultivation conditions impacts the quality of black tea. While with lower leaf grades, floral fragrance first declined before intensifying, sweet scents continuously decreased throughout grade reduction from bud to third leaf (Xiao et al., 2025). Similarly, the integration of e-tongue data and trained sensory evaluation demonstrated that black tea processed with a single bud was sweeter, fresher, fruity, and less bitter than one bud and two leaves (R. Chen et al., 2024). In addition, shading, as an important agricultural measure to enhance tea quality, can significantly increase the content of free amino acids and reduce that of tea polyphenols, thereby improving tea freshness and briskness (Hu et al., 2024; Niu et al., 2025). The exploration and documentation of traditional knowledge regarding wild and regional plant resources have long provided inspiration for functional food development and phytotherapy (Khatun et al., 2012; Korkmaz et al., 2016). Comparative metabolomic analysis reveals distinct profiles between wild and domesticated tea varieties. Notably, terrace-grown tea exhibits a significant enrichment in L-glutamic acid, L-proline, D-valine, and EGCG. These metabolites are related to umami, astringency, and bitterness, ultimately yielding a more intense and stronger flavor profile in cultivated samples than in natural tea varieties (Wang et al., 2025). A systematic assessment of Juhong tea's nonvolatile compounds, taste, color, and antioxidant capacity will help address diverse consumer flavor preferences and guide targeted citrus tea product development.

Modern sensory assessment technologies, such as electronic tongues, electronic nose, and color difference analysis, are progressively transforming sensory evaluations, as traditional methods were susceptible to subjective interference (Sheng et al., 2024; Sun et al., 2023; Wei et al., 2025; Zan et al., 2025). While numerous studies have independently investigated the metabolite variations of individual citrus peel varieties and the characteristics of diverse black tea sources, the effects of their combination, specifically how different black tea raw materials impact the comprehensive quality characteristics of Juhong tea, remain largely unclear. This study aims to systematically evaluate and compare four types of Juhong tea prepared from distinct black tea sources: traditional one-bud-two-leaf Juhong tea (TJH), single-bud Juhong tea (BJH), wild Juhong tea (WJH), and shaded Juhong tea (SJH). The specific research objectives are: (1) to characterize the sensory profiles, electronic-tongue taste properties, and color parameters of Juhong teas using multi-sensor evaluation; (2) to quantify core taste-active constituents, including free amino acids, catechins, theaflavins, and soluble sugars; (3) to profile and identify non-volatile differential metabolites using widely targeted metabolomics via UHPLC-MS/MS; and (4) to evaluate in vitro antioxidant capacities (DPPH, T-AOC, and HSA) and establish clear correlation networks between key chemical constituents and functional attributes. Ultimately, this work seeks to uncover the chemical basis of Juhong tea flavor formation and provide a robust theoretical foundation for raw material selection, quality control, and processing optimization in citrus-tea composite manufacturing.”

## 2 Material and methods

### 2.1 Preparation of Juhong tea samples

Fresh leaf materials were collected from tea trees of the E'cha 1 (*Camellia sinensis* L.) except for WJH. Tea trees were planted in Longma Village, Longfeng Town, Enshi City, Hubei Province. For fresh SJH leaves, environmental shading was achieved by installing nets with approximately 90% opacity 15 cm above the canopy apex. This light-deprivation was maintained for three weeks. For WJH, tea trees were cultivated without human intervention for 10 years. Regarding the raw materials, only BJH samples were harvested as single buds, whereas the remaining three samples comprised one bud with two leaves. All fresh leaves were simultaneously collected on April 16, 2024, and then processed using a standardized procedure for black tea. Processing parameters are described below. Fresh leaves were first withered for 12 h at room temperature before being rolled for 1.5 h using a light-heavy-light pressure pattern. Then, the rolled leaves were fermented at 30 °C and 90% humidity for 5 h, followed by oven-drying at 120 °C for 20 min and then drying at 80 °C for 2.5 h. Finally, black teas were stored at 4 °C until further use. Juhong teas were processed in November 2024 using the following specific procedure (Fig. S1). First, high-quality ‘Nanfeng’ tangerine (*Citrus reticulata* Blanco) of 4.5–5.0 cm in diameter were selected and washed. The pulp was removed, and the peels were thoroughly cleaned. After initial drying, each hollow peel was filled with 6 g black tea and resealed. The stuffed peels were then sequentially dried, first at 100 °C for 1 h and then at 60 °C for 3 h, until the final moisture content was ≤7.0%.

### 2.2 Chemicals

The chemicals used in this study are listed in Table S1.

### 2.3 Evaluation of sensory qualities

For conventional sensory scoring, each Juhong tea sample was brewed with 150 mL boiling water for 5 min. After filtering the tea liquor, five key attributes, namely appearance, aroma, taste, infusion color, and infusion leaves, were scored on a scale of 0 to 10. Each sample was evaluated in triplicate. To ensure blinding, every cup was marked with a random three-digit code, and the serving order was randomized among panelists. All participants provided informed consent before the sensory evaluation and could withdraw at any time without a reason. The rights and privacy of all participants were maintained during all experiments. The products tested were safe for consumption.

### 2.4 Electronic tongue for taste evaluation and infusion chromatic difference analysis

Tea powder (3 g) was added to a tasting cup and brewed with 150 mL of boiling water for 5 min. The mixture was then filtered through gauze. A 100 mL aliquot of the filtrate was equilibrated to 25 °C before analysis. The electronic tongue system (TS-5000Z, INSENT, Japan), equipped with six chemical sensors, was used to detect umami, saltiness, sourness, bitterness, astringency, and sweetness. A reference electrode containing 30 mM potassium chloride and 0.3 mM tartaric acid was used. Taste intensity was expressed in Weber-Fechner units. Simultaneously, color parameters of the remaining filtrate were measured using a colorimetric cell with a CM-5 spectrophotometer (Konica Minolta, Japan). Each sample was prepared and analyzed in triplicate.

### 2.5 Determination of main taste components

Free amino acid quantification was conducted following the protocol described in reference with minor changes (Feng et al., 2020). For extraction, 0.1 g of the powdered sample was immersed in 10 mL of deionized water and heated in a boiling water bath (100 °C) for 30 min. Following centrifugation (1307 ×*g*, 10 min, 4 °C), the obtained supernatant was transferred and adjusted to a total volume of 10 mL. Before analysis, the extract was filtered through a 0.22 μm membrane filter. Chromatographic separation was performed on a Waters AccQ.Tag C18 column (3.9 mm × 150 mm, 4 μm) using a Waters 2695 HPLC platform. For quantification, an external standard method was employed, and all analyses were executed in triplicate.

The determination of aqueous extract followed GB/T 8305–2013 tea -determination of aqueous extract with minor modifications. A total of 0.6 g of tea powder was soaked in 80 mL boiling water, transfer to a 100 °C water bath for 45 min, and then filtered. The volume was subsequently adjusted to 100 mL. After placing the filter paper and tea residue in an aluminum box and drying it at 120 °C for 1 h, they were taken out to cool down and then dried for another 1 h to calculate the weight of the tea residue. The filtrate was then used for the determination of free amino acids, soluble sugars, and flavonoids. The total flavonoid content was determined using the aluminum chloride colorimetric method at 420 nm. Soluble sugar content was determined by anthrone‑sulfuric acid colorimetry, and the absorbance was measured at 620 nm using a standard curve prepared with anhydrous glucose. Soluble protein content was determined using Coomassie brilliant blue according to manufacturer's instructions (Jiancheng, Nanjing, China).

The Folin-Ciocalteu colorimetric assay was used to determine the total tea polyphenol (TP) content. Specifically, 0.20 g of tea powder was extracted with 5 mL of 70% aqueous methanol at 70 °C for 10 min, during which the mixture was vortexed every 5 min. After cooling and centrifugation at 3500 rpm for 10 min, the supernatant was collected. This extraction was repeated twice and the combined fractions were adjusted to a final volume of 10 mL using methanol. Quantification was performed using gallic acid as a reference standard. Caffeine and catechin were analyzed using HPLC (Waters Alliance e2695, Waters Technologies Inc., Milford, MA, USA) as previously reported (Huang et al., 2024). Theaflavin components were determined via HPLC following the methodology described by Ai et al. (2024). All experiments were performed in triplicate for each group.

### 2.6 Antioxidant ability analysis

The antioxidant potential of four Juhong teas was assessed through three in vitro assays: 2,2-diphenyl-1-picrylhydrazyl free radical scavenging ability (DPPH), total antioxidant capacity (T-AOC), and hydroxyl free radical scavenging activity (HSA). For T-AOC determination, 1 g of sample was homogenized on ice with 9 mL of 0.9% sodium chloride solution, followed by centrifugation at 2500 rpm for 10 min. The supernatant was collected and assayed using T-AOC reagent kits (Jiancheng, Nanjing, China) according to the manufacturer's instructions. For DPPH assay, 0.1 g of sample was homogenized on ice with 2 mL of 80% methanol and centrifuged at 12000 rpm for 10 min. Then, 1 mL of supernatant was diluted with 3 mL of 80% methanol, and the DPPH radical scavenging activity was subsequently determined using the appropriate reagent kit (Jiancheng, Nanjing, China) according to manufacturer's instructions. For HSA detection, the sample (0.1 g) was dissolved in 1 mL of distilled water, followed by sequential grinding on ice and homogenization. The extract was then clarified by centrifugation at 12,000 r/min for 10 min at 4 °C, and the supernatant was assayed using a reagent kit (Acmec Biochemical Co., Ltd., Shanghai, China) according to the manufacturer's instructions.

### 2.7 Widely targeted metabolomics analysis by UHPLC-MS/MS

Every five Juhong teas were mixed and ground together to form one biological replicate. Samples were pulverized using a mixer mill (MM 400, Retsch) with a zirconia bead at 30 Hz for 30 s. Subsequently, 100 mg of lyophilized powder was extracted with 1.2 mL of a 70% aqueous methanol solution. The mixture was vortexed for 30 s every 30 min for a total of six cycles, followed by overnight incubation at 4 °C. After centrifugation at 12,000 rpm for 10 min, the resulting supernatants were filtered through a 0.22 μm microporous membrane before UHPLC-MS/MS analysis.

The UHPLC analysis was conducted on a Shimadzu UHPLC system (CBM-20A controller) coupled to a QTRAP® 4500+ mass spectrometer. Chromatographic separation was achieved on a Waters ACQUITY UPLC HSS T3 C18 column (1.8 μm, 2.1 × 100 mm) maintained at 40 °C. The mobile phase, consisting of 0.1% formic acid in water (A) and 0.1% formic acid in acetonitrile (B), was injected at 0.4 mL/min at a volume of 2 μL. The gradient elution program was set as follows: initially 95% A and 5% B, linearly changed to 5% A and 95% B over 0–10.0 min, held until 11.0 min, back to 95% A and 5% B at 11.1 min, and maintained until 15.0 min.

Mass data were collected using an AB4500+ Q TRAP UPLC-MS/MS system equipped with an ESI Turbo Ion-Spray source, operating under both positive and negative ionization modes. The source parameters were set as follows: source temperature at 550 °C; ion-spray voltages at +5500 V (positive) and − 4500 V (negative); curtain gas, nebulizer gas, and heater gas pressures at 25.0, 50, and 60 psi, respectively; and high collision-activated dissociation (CAD). Multiple reaction monitoring (MRM) modes with nitrogen as the collision gas were employed for quantification, with declustering potentials and collision energies optimized for each metabolite.

To monitor experimental repeatability and ensure the reliability of the data, quality control (QC) samples, prepared by mixing equal aliquots of extracts from all samples, were injected at regular intervals throughout the analytical run. Total peak area normalization was applied to the raw dataset to minimize inter-batch variations, and metabolites exhibiting a relative standard deviation (RSD) > 30% across QC samples were removed.

### 2.8 Statistical data analysis

For metabolomic analyses, principal component analysis (PCA) and orthogonal partial least squares discriminant analysis (OPLS-DA) of the metabolites were performed using the SIMCA 14.1(Sartorius, Göttingen, Germany). Significantly different metabolites among samples were screened using variable importance in projection (VIP > 1), *p*-value <0.05 and |log₂FC| > 1. Venn diagrams and UpSet plots were generated using the VennDiagram and UpSetR packages in R (version 4.3.0). Volcano plots were constructed for each comparison using the ggplot2 package. Heatmap visualization was generated using TBtools to display metabolite expression patterns. For univariate analysis, inter-group comparisons and pairwise correlation analyses were performed using GraphPad Prism 7 (Dotmatics, CA, USA). One-way ANOVA followed by Tukey's test was used for multiple comparisons, and pearson correlation analysis was used to assess associations among variables, with a significance threshold of *p* < 0.05. Results are expressed as the mean ± SD based on three independent replicates.

## 3 Results and discussion

### 3.1 Sensory evaluation and electronic tongue analysis of four Juhong teas

The appearance and sensory characteristics of the four Juhong teas were systematically evaluated using morphological observation, traditional sensory scoring by quantitative descriptive analysis, chromatic difference analysis, and electronic tongue analysis (Fig. 1). The infusion appearance and color parameters of Juhong teas are shown in Fig. 1 A, B. The L*, a*, and b* values denote brightness, redness (+)/greenness (−), and yellowness (+)/blueness (−), respectively. SJH exhibited the highest L* value, which was significantly higher than those of TJH, WJH, and BJH (*p* < 0.001). Regarding a* and b*, TJH showed significantly red and yellow values than SJH,with average values of 15.37 and 83.08, compared to 1.46 and 48.12 for SJH (*p* < 0.0001). Overall, TJH demonstrated the most saturated coloration, with high a* and b* values, suggesting a higher degree of pigmentation, whereas SJH showed the highest lightness but lower chromatic intensity.

**Fig. 1:**
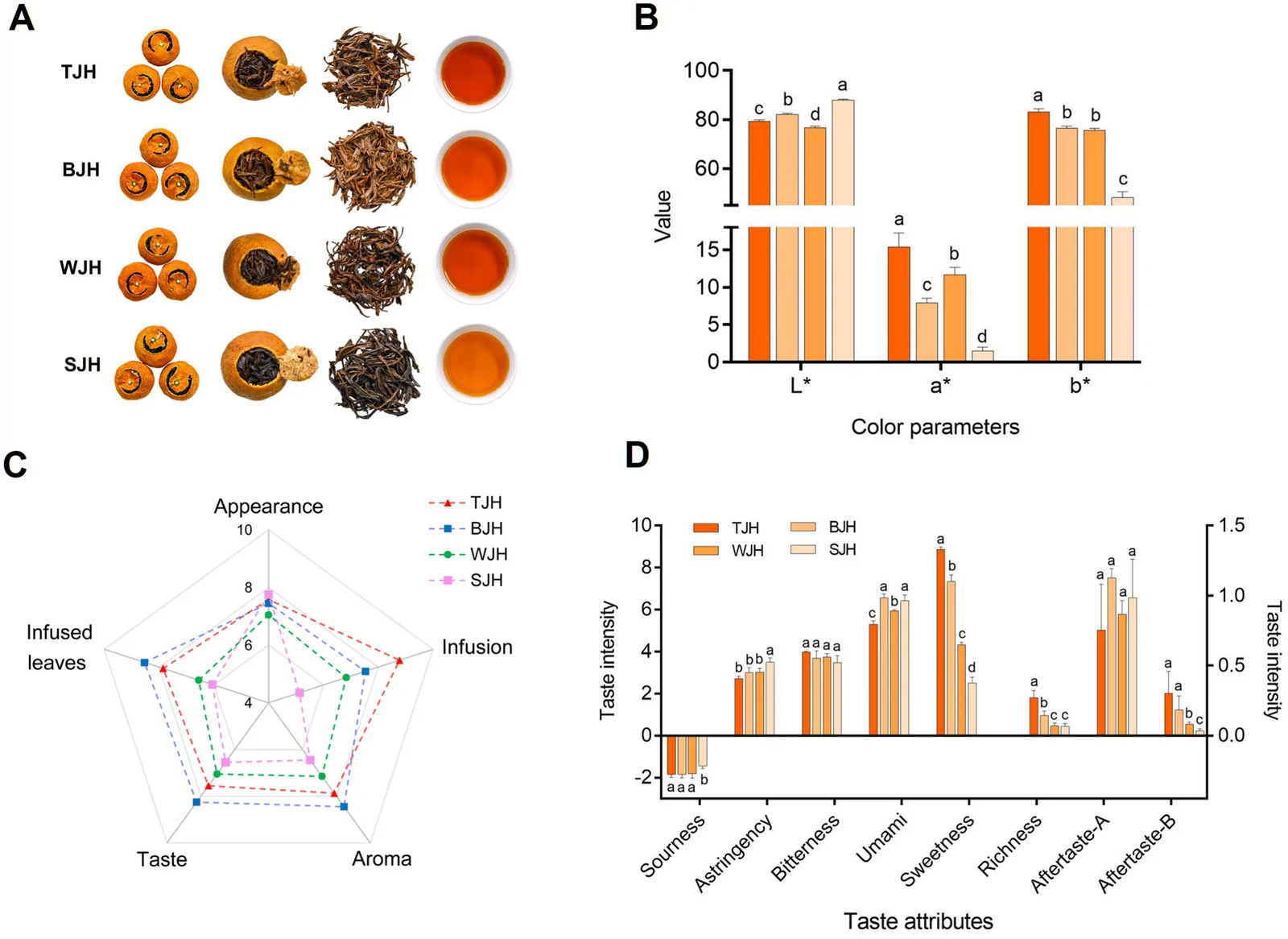
Sensory, quantitative descriptive analysis, and electronic tongue results of four Juhong teas. (A) Appearance, infused leaves, and soup color. (B) Infusion color parameters. (C) Scores of sensory quality evaluation. (D) Electronic tongue analysis, the intensities of sourness, astringency, bitterness, umami, and sweetness are plotted against the primary left y-axis, while richness, aftertaste-A, and aftertaste-B are plotted against the secondary right y-axis. Data are presented as mean ± SD. Bars labeled with different lowercase letters represent significant intersample differences (*p* < 0.05, Tukey's test). TJH, traditional Juhong; BJH, single bud Juhong; WJH, wild Juhong; SJH, shaded Juhong.

Sensory evaluation revealed significant differences among the four samples in most quality attributes, except for appearance, which showed no significant differences (*p* > 0.05) (Table S2). TJH obtained the highest infusion score (8.98), which was significantly higher than those of BJH (*p* = 0.0039), WJH (*p* = 0.0087), and SJH (*p* = 0.0017). For aroma and taste, BJH achieved the highest scores (8.43 and 8.24, respectively), significantly outperforming WJH and SJH (*p* < 0.05), while TJH exhibited intermediate scores with no significant difference from BJH (*p* > 0.05). Overall, BJH demonstrated superior quality in aroma, taste, and infused leaves, achieving the highest overall sensory score.

The electronic tongue analysis (Fig. 1D) further distinguished the taste attributes among the samples. TJH, BJH, and WJH showed no significant differences in sourness, astringency, aftertaste-A, or bitterness (*p* > 0.05). TJH exhibited higher scores in sweetness (8.85) and richness (0.27), contributing to a more balanced flavor profile. Conversely, SJH showed the lowest sweetness (2.51) and richness (0.07), but the highest umami (6.42) and a slight increase in astringency (3.49). These sensory attributes suggest that shading inhibited photosynthesis, suppressing soluble sugar synthesis while altering amino acid metabolism, which distinctively shifted the taste profile from sweet-dominant to umami-rich.

### 3.2 Main taste component analysis of the four Juhong teas

The flavor profile of tea results from comprehensive interactions among various internal constituents (Jiang et al., 2019). Amino acids significantly influence sensory characteristics, serving as foundation for umami, sweet, and bitter flavor notes. As detailed in Table 1, absolute quantification identified 17 distinct amino acids, categorized into umami (3), sweet (5), and bitter (7) groups, along with two others based on their gustatory properties (Geng et al., 2026). Among the four Juhong teas, BJH displayed the most substantial concentration of umami-contributing amino acids (20.56 mg/g), followed by SJH (19.26 mg/g), which were both significantly higher than TJH (10.21 mg/g) and WJH (5.45 mg/g) (*p* < 0.05). Theanine (Thea) was the predominant umami component, accounting for 86%–89% of the total umami amino acids. Its content varied significantly among samples, with BJH (18.38 mg/g) and SJH (16.62 mg/g) showing the highest levels, followed by TJH (9.09 mg/g) and WJH (4.74 mg/g) (*p* < 0.05). The sweet amino acid fraction, primarily comprising alanine (Ala), threonine (Thr), and serine (Ser). BJH, TJH, and SJH accumulated significantly higher levels of sweet amino acids (5.42, 4.60, and 4.21 mg/g, respectively) than WJH (1.05 mg/g) (*p* < 0.05). Among them, Ala was the most abundant sweet component across all samples, peaking at 2.82 mg/g in SJH. Although bitter amino acids were the most diverse group, their cumulative content remained relatively low BJH exhibited the highest total bitter amino acid content (6.15 mg/g), followed by SJH (4.75 mg/g) and TJH (3.96 mg/g), all of which were significantly higher than that of WJH (2.23 mg/g) (*p* < 0.05). In terms of total amino acid (TAA) content, BJH yielded the highest value (32.69 mg/g), followed closely by SJH (28.88 mg/g), and both were significantly higher than TJH (19.29 mg/g) and WJH (9.11 mg/g) (*p* < 0.05). Notably, the elevated TAA and theanine levels in the shaded sample (SJH) align with previous studies demonstrating that shading significantly enhances free amino acid accumulation by suppressing their catabolism and promoting nitrogen assimilation (Niu et al., 2025).

**Table 1:** Content of amino acids in four Juhong tea samples.

Name	Taste	Contents (mg/g)			
		TJH	BJH	WJH	SJH
Asp	Umami	0.29 ± 0.07b	0.37 ± 0.02b	0.19 ± 0.13b	0.51 ± 0.06a
Glu		0.84 ± 0.07b	1.81 ± 0.08a	0.51 ± 0.06c	2.12 ± 0.39a
Thea		9.09 ± 0.43b	18.38 ± 0.16a	4.74 ± 0.81c	16.62 ± 2.07a
Total		10.21 ± 0.37b	20.56 ± 0.14a	5.45 ± 0.99c	19.26 ± 2.54a
Ser	Sweetness	0.49 ± 0.02b	0.68 ± 0.02a	0.11 ± 0.08c	0.49 ± 0.08b
Gly		0.22 ± 0.01a	0.23 ± 0.02a	0.11 ± 0.02b	0.21 ± 0.01a
Thr		0.69 ± 0.05b	1.47 ± 0.28a	0.13 ± 0.02c	nd
Ala		2.33 ± 0.40a	2.40 ± 0.31a	0.39 ± 0.03b	2.82 ± 0.55a
Pro		0.88 ± 0.03a	0.65 ± 0.01b	0.30 ± 0.14c	0.69 ± 0.10b
Total		4.60 ± 0.45a	5.42 ± 0.58a	1.05 ± 0.09b	4.21 ± 0.67a
Arg	Bitterness	0.07 ± 0.03c	0.32 ± 0.01b	0.00 ± 0.00d	1.00 ± 0.23a
Tyr		0.63 ± 0.02b	0.80 ± 0.07a	0.39 ± 0.07d	0.58 ± 0.01c
Val		0.37 ± 0.04a	0.34 ± 0.01a	0.05 ± 0.05c	0.26 ± 0.03b
Lys		0.51 ± 0.06b	0.79 ± 0.05a	0.25 ± 0.01c	0.49 ± 0.10b
Ile		0.63 ± 0.02b	0.99 ± 0.01a	0.40 ± 0.01c	0.64 ± 0.08b
Leu		0.60 ± 0.32b	1.64 ± 0.05a	0.42 ± 0.01b	0.85 ± 0.12b
Phe		1.16 ± 0.07a	1.26 ± 0.07a	0.71 ± 0.03c	0.92 ± 0.04b
Total		3.96 ± 0.41b	6.15 ± 0.10a	2.23 ± 0.12c	4.75 ± 0.47b
His		0.11 ± 0.00b	0.34 ± 0.02a	0.19 ± 0.19b	0.21 ± 0.03b
Met		0.42 ± 0.01a	0.22 ± 0.04b	0.20 ± 0.03b	0.45 ± 0.23a
TAAs		19.29 ± 1.20b	32.69 ± 0.65a	9.11 ± 1.17c	28.88 ± 3.47a

Note: The data were expressed as mean value ± standard deviation (mean ± SD). Different lowercase letters in a row indicate significant differences between samples (*p* < 0.05, Tukey's test). TAA, Total free amino acids, TJH, traditional Juhong; BJH, single bud Juhong; WJH, wild Juhong; SJH, shaded Juhong.

As shown in Fig. 2A, the aqueous extract proportions were similar across all four samples, whereas electronic tongue analysis indicated notable differences in richness. This suggests that flavor development may depend on complex interactions among compounds such as sugars, amino acids, and polyphenols. Tea polyphenols (TPs), the main bioactive compounds in tea plants, largely determine the flavor profile and overall quality attributes of tea (L. Zhang et al., 2019). TP content was significantly enriched in BJH (44.61 mg/g) (*p* < 0.05); In contrast, the shaded sample (SJH) accumulated the lowest TP content (20 mg/g). This finding aligns with prior studies suggesting that shading significantly suppresses polyphenol biosynthesis and reduces its accumulation in tea leaves (Hu et al., 2024). As the outer shell of Juhong tea, the tangerine peels are rich in flavonoids and inevitably migrate into the tea infusion during brewing. During black tea fermentation, flavonoids undergo significant degradation, reducing astringency and bitterness while enhancing the mellowness of the black tea. (Jiang et al., 2019). In this study, however, SJH exhibited the highest flavonoid content (13.5 mg/g), with SJH being more than 2-fold higher than that of BJH (6 mg/g) (*p* < 0.05). This disparity in flavonoid levels may account for the divergent sensory profiles of these Juhong teas. The elevated flavonoid concentration in SJH potentially contributes to its more pronounced astringent taste compared to the other three samples, as well as enhanced liquor brightness.

**Fig. 2:**
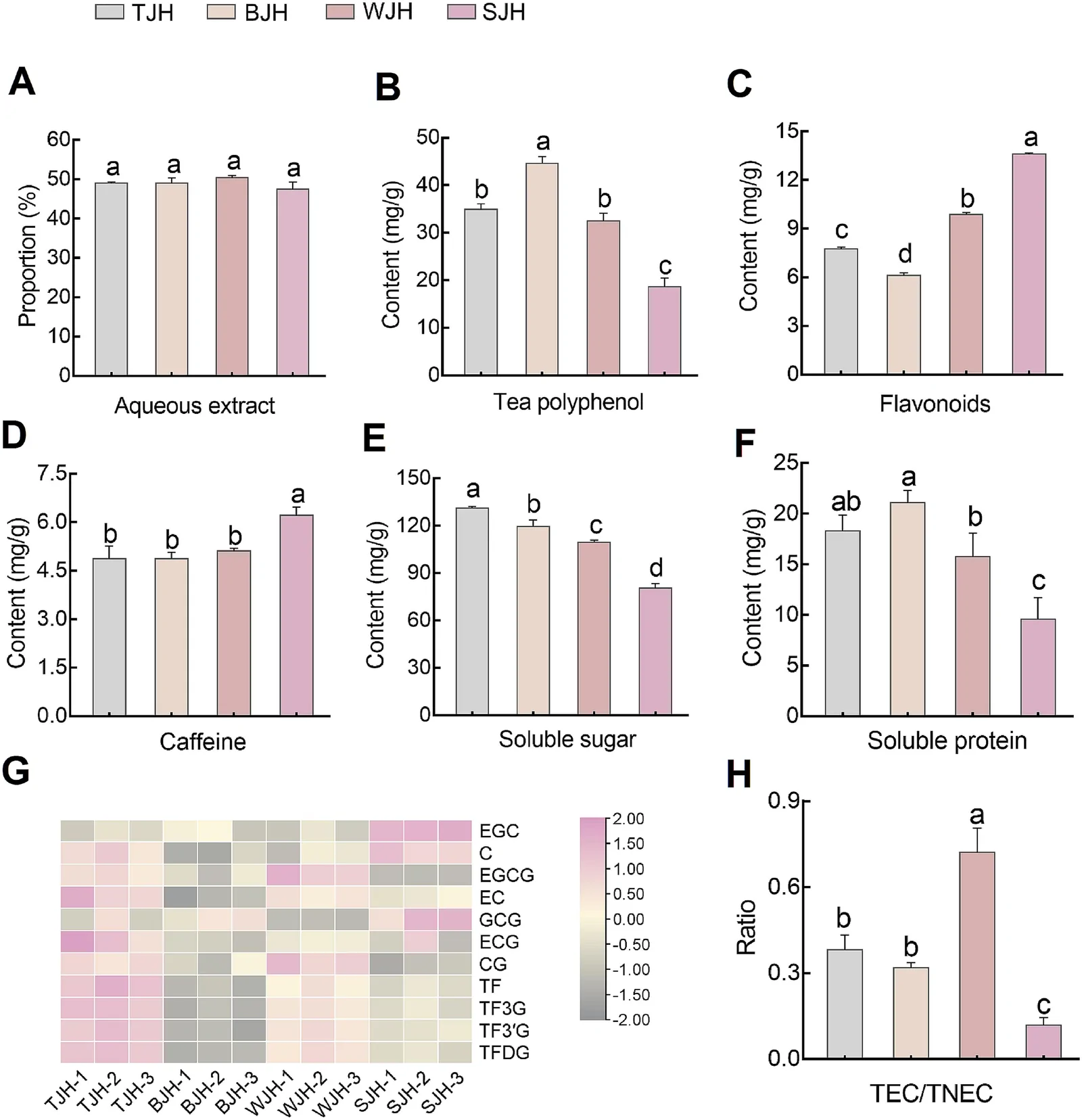
Differences in main taste components in the four Juhong tea samples. (A) Aqueous extract proportion, (B) Tea polyphenol content, (C) Flavonoid content, (D) Caffeine content, (E) Soluble sugar content, (F) Soluble protein content, (G) Heatmap of individual catechin components and theaflavins, (H)TEC/TNEC ratio. Different lowercase letters (a–d) within a single row denote significant disparities among the four Juhong tea varieties, as determined by Tukey's multiple range test (*p* < 0.05). TPs, tea polyphenol; CAF, caffeine; EGCG, epigallocatechin gallate; GCG, gallocatechin gallate; ECG, epicatechin gallate; CG, catechin gallate; EGC, epigallocatechin; C, catechin; EC, epicatechin; TEC, total ester catechins; TNEC, total nonester catechins; TEC/TNEC, The ratio of TEC/TNEC; TF, theaflavin; TF3G, theaflavin-3-gallate; TF3’G, theaflavin-3′-gallate; TFDG, theaflavin-3,3′ -gallate.

Soluble sugars are the major contributors to the sweetness and thickness of tea infusions. Moreover, they play a pivotal role in modulating bitterness and astringency, thereby refining the overall sensory profile by balancing taste attributes (Fang et al., 2024; Zhang et al., 2026). Previous studies have demonstrated that soluble monosaccharides and sugar alcohols such as d-glucose, inositol, d-fructose, and D-galactose, are significantly enriched in premium flavored products like white and black teas, serve as the core material basis for establishing the exceptional sweetness profile of tea infusions (Xing et al., 2026). The soluble sugar content showed a significant decreasing trend: TJH > BJH > WJH > SJH (*p* < 0.05), which was consistent with the electronic tongue results regarding sweetness. Soluble proteins critically affect black tea quality by interacting with polyphenols to influence cream formation, liquor clarity, and taste balance (Yu et al., 2020). SJH was significantly lower than the other three samples (*p* < 0.05), indicating that shading severely restricts protein biosynthesis, likely due to reduced photosynthetic carbon skeletons. Furthermore, BJH exhibited the highest soluble protein content, significantly higher than WJH (*p* = 0.0329) The caffeine-theaflavin complex simultaneously activates both bitter and umami taste receptors, thereby imparting black tea with a robust liquor and brisk, refreshing, and distinctly lively characteristics (Shen et al., 2025). Previous research indicated that caffeine content was significantly increased under partial or full shading (Liu et al., 2025). In this study, the caffeine content was highest in SJH (6.21 mg/g) and was significantly higher than that of TJH (4.87 mg/g, *p* = 0.0323), BJH (4.88 mg/g, *p* = 0.0020), and WJH (5.11 mg/g, *p* = 0.0308). However, no significant differences were observed among the latter three samples (*p* > 0.05). Catechins are the most abundant flavonoids in the tea leaves. During black tea processing, they undergo oxidation and polymerization to form theaflavins, thearubigins, theabrownins, and other high-molecular-weight polymers, which largely determine the taste and color of black tea (Fan et al., 2016). The ester-type catechins identified in this study included epigallocatechin gallate (EGCG), gallocatechin gallate (GCG), epicatechin gallate (ECG), and catechin gallate (CG), whereas nonester catechins consisted of epigallocatechin (EGC), catechin (C), and epicatechin (EC). The ratio of ester-type to nonester-type catechin directly determines the bitterness–mellowness balance of black tea. WJH exhibited the highest TEC/TNEC ratio (0.72) (Fig. 2H), significantly higher than TJH (0.38, *p* = 0.0002), BJH (0.32, *p* < 0.0001), and SJH (0.12, *p* < 0.0001). Theaflavins are formed by enzymatic oxidation and polymerization of catechins. As prominent bioactive pigments and polyphenolic constituents in black tea, theaflavins (TFs) act as potent natural antioxidants with various health-promoting properties. (Jin et al., 2024; Shen et al., 2025). Furthermore, TFs are strongly associated with with brew richness and serve as primary determinants of its vibrant orange-yellow hue. Notably, these compounds are indispensable for the formation of the characteristic “golden ring,” a key indicator of high-quality black tea infusions (Dong et al., 2018; Wang et al., 2014). Of the four Juhong teas, all three theaflavin monomers (TF3G, TF3’G, and TFDG) exhibited a consistent decreasing trend: TJH > WJH > SJH > BJH (*p* < 0.05, Fig. 2G). Notably, TFDG was the most abundant monomer across all samples, with TJH showing the highest level. This significant accumulation of theaflavins, particularly TFDG, likely contributed to the vibrant orange-yellow hue and the brisk, bright taste of TJH.

### 3.3 Profiling of nonvolatile metabolites

#### 3.3.1 Systematic evaluation of nonvolatile metabolomic profiles

TJH, BJH, WJH, and SJH were used as four different experimental groups, with three biological replicates for each group. A total of 520 nonvolatile metabolites, distributed across 13 categories, were initially identified in the four Juhong tea samples (Table S3). These categories included 109 flavonoids, 76 alkaloids, 60 amino acids and their derivatives, 42 phenolic acids, 37 lipids, 33 lignans and coumarins, 31 terpenoids, 31 nucleotides and their derivatives, 27 organic acids, 9 sugars and alcohols, 6 stilbenes, 2 tannins, and 57 other metabolites (Fig. 3A). Flavonoids, alkaloids, amino acid derivatives, and phenolic acids emerged as major nonvolatile constituents in Juhong teas sourced from diverse black tea cultivars. The intersample variation is partially visualized in the heatmap (Fig. 3B), which reveals metabolites enriched in one Juhong tea type but absent in the remaining three.

**Fig. 3:**
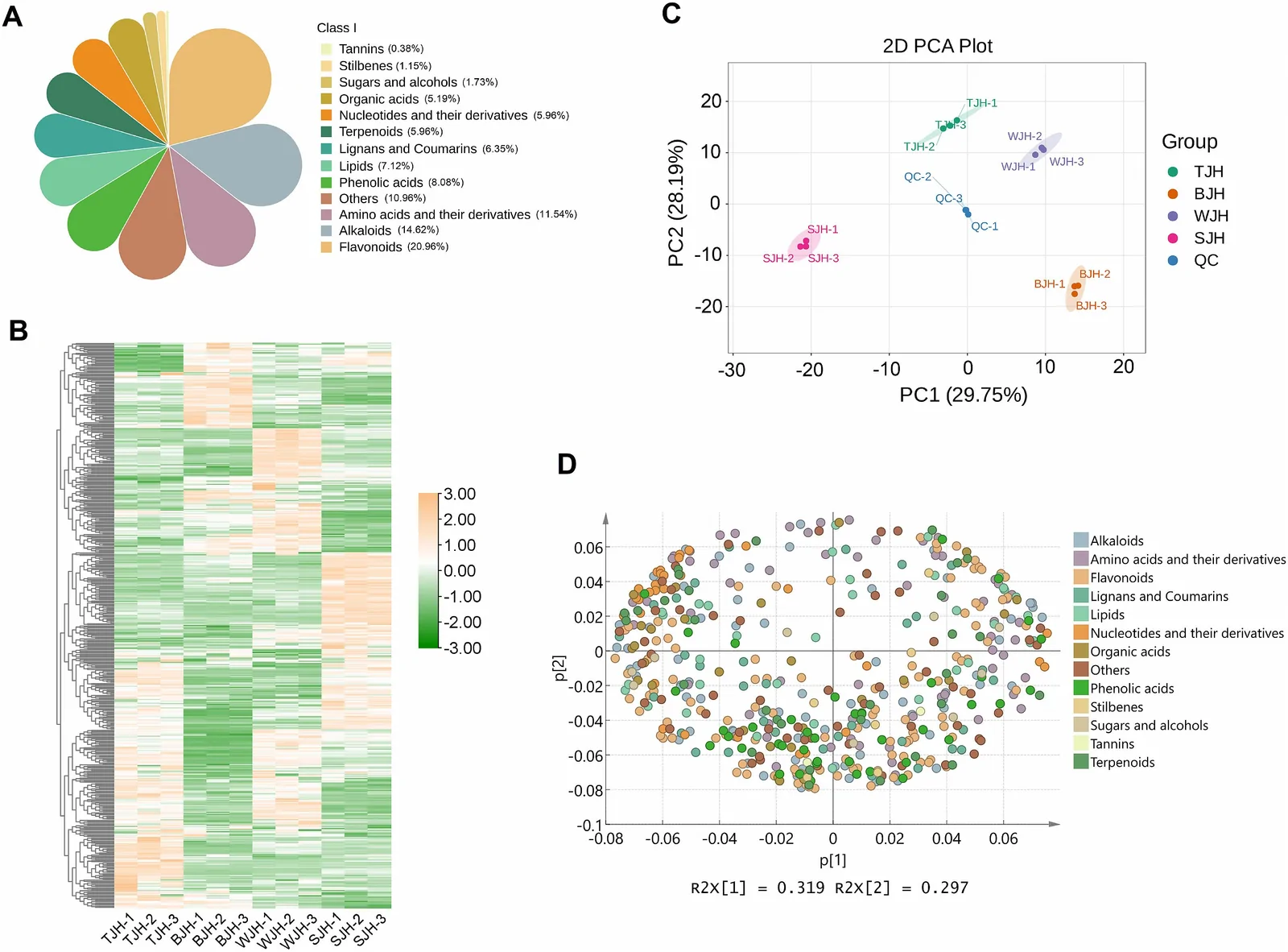
Metabolic profiling and multivariate statistical analysis of the four Juhong tea samples. (A) Classification of the 520 identified metabolites. (B) Hierarchical cluster analysis (HCA) heatmap of the metabolites across all samples. The color scale indicates the relative abundance of metabolites (green, low; orange, high). (C) Principal component analysis (PCA) score plot based on the metabolic profiles. (D) Loading plot (p[1] vs. p[2]) of PCA. TJH, traditional Juhong; BJH, single bud Juhong; WJH, wild Juhong; SJH, shaded Juhong; QC, quality control. Metabolic profiling and multivariate statistical analysis of the four Juhong tea samples. (A) Classification of the 520 identified metabolites. (B) Hierarchical cluster analysis (HCA) heatmap of the metabolites across all samples. The color scale indicates the relative abundance of metabolites (green, low; orange, high). (C) Principal component analysis (PCA) score plot based on the metabolic profiles. (D) Loading plot (p[1] vs. p[2]) of PCA. TJH, traditional Juhong; BJH, single bud Juhong; WJH, wild Juhong; SJH, shaded Juhong. QC, quality control. (For interpretation of the references to color in this figure legend, the reader is referred to the web version of this article.)

To clarify overall metabolomic divergence, principal component analysis (PCA) was conducted (Fig. 3C). The PCA score plot showed that PC1 and PC2 explained 29.75% and 28.19% of the total variance, respectively. The four Juhong tea samples exhibited distinct separation trends, while the quality control (QC) samples tightly clustered together, confirming excellent signal stability and analytical reproducibility. Furthermore, the loading plot (Fig. 3D) revealed that flavonoids, alkaloids, and amino acids were the predominant contributors to the metabolic divergence among the four groups (R^2^X[1] = 0.319, R^2^X[2] = 0.297).

To further pinpoint the specific chemical markers driving the metabolic divergence, the top 30 contributing variables in the PCA model were screened based on their Euclidean distances (Table S4). Flavonoids were the largest single class, accounting for 36.7%. Specifically, flavonoids such as quercetin-3-O-α-L-rhamnopyranoside (p[1] = −0.0768), 6-methoxy-quercetin-7-O-glucoside, and apigenin-7-O-glucoside exhibited high loading values, indicating their dominant roles in the discrimination of the four Juhong tea samples. The Euclidean distances of these top 30 metabolites ranged narrowly from 0.0769 to 0.0788, suggesting that the overall metabolic divergence was driven by a coordinated network of multiple chemical classes rather than a single compound.

#### 3.3.2 Comparative analysis of differential nonvolatile metabolites

OPLS-DA is a supervised multivariate statistical method that uses predefined group labels to evaluate contribution of individual variables to class discrimination. This approach provides additional insights into how the black tea raw material influence the nonvolatile metabolite profile of Juhong teas. Here, supervised OPLS-DA models were constructed to identify the key discriminant metabolites among the four Juhong tea samples. Model performance was evaluated by 200-permutation cross-validation; negative Q2 intercepts confirmed the absence of overfitting (Fig. S3). Pairwise models BJH vs. TJH (R^2^Y = 1, Q^2^ = 0.984, *p* < 0.005), SJH vs. TJH (R^2^Y = 1, Q^2^ = 0.976, *p* < 0.005), and WJH vs. TJH (R^2^Y = 1, Q^2^ = 0.971, *p* < 0.005) exhibited high explained variance and strong predictive power.

Significantly different metabolites among Juhong tea samples were screened using variable importance in projection (VIP) > 1, *p*-value <0.05 and |log₂FC| > 1. Venn analysis revealed shared and unique differential metabolites among the three pairwise comparisons. Twenty-six compounds were commonly dysregulated in BJH vs. TJH, SJH vs. TJH, and WJH vs. TJH. In contrast, 63, 73, and 27 unique differential metabolites were identified in BJH vs. TJH, SJH vs. TJH, and WJH vs. TJH, respectively (Fig. 4A). Pairwise comparisons revealed 156 differential metabolites (39 upregulated and 117 downregulated) for BJH vs. TJH, 158 (90 upregulated and 68 downregulated) for SJH vs. TJH, and 89 (36 upregulated and 53 downregulated) for WJH vs. TJH (Table S5, Fig. 4B). Advanced Pie plot (Fig. 4B) and Volcano plots (Fig. 4C) were performed to analyze the distinct effects of black tea variety on secondary metabolites of Juhong tea in-depth. Among all secondary metabolites, flavonoids exhibited the most pronounced changes (Fig. 4D). Compared with TJH, quercetol C showed the greatest downregulation in BJH, with an |log_2_FC| value of 10.79, followed by an alkaloid (isocorydine), a lipid (lysoPC20:0(sn-2)), an organic acid (constictic acid), and a flavonoid (apigenin7-O-glucoside), all with |log_2_FC| > 4.0. Conversely, two flavonoids (naringenin-7-O-glucoside, 5,7-dihydroxy-2-(2,3,4-trihydroxyphenyl) chromen-4-one), two amino acids and their derivatives (glutamic acid, l-glutamine), and an alkaloid (chelerythrine) exhibited the greatest upregulation, with |log_2_FC| > 2 compared with TJH. In SJH, one flavonoid (2’-*O*-methylisoliquiritigenin) and one phenolic acid (1-(4-methoxyphenyl) propane-1,2-diol) exhibited the greatest downregulation, with |log_2_FC| > 7 compared with TJH. In WJH, acoric acid and isocorydine both exhibited |log_2_FC| > 9 compared with TJH. All these metabolites are the primary drivers of the quality differences observed among the four black tea types.

**Fig. 4:**
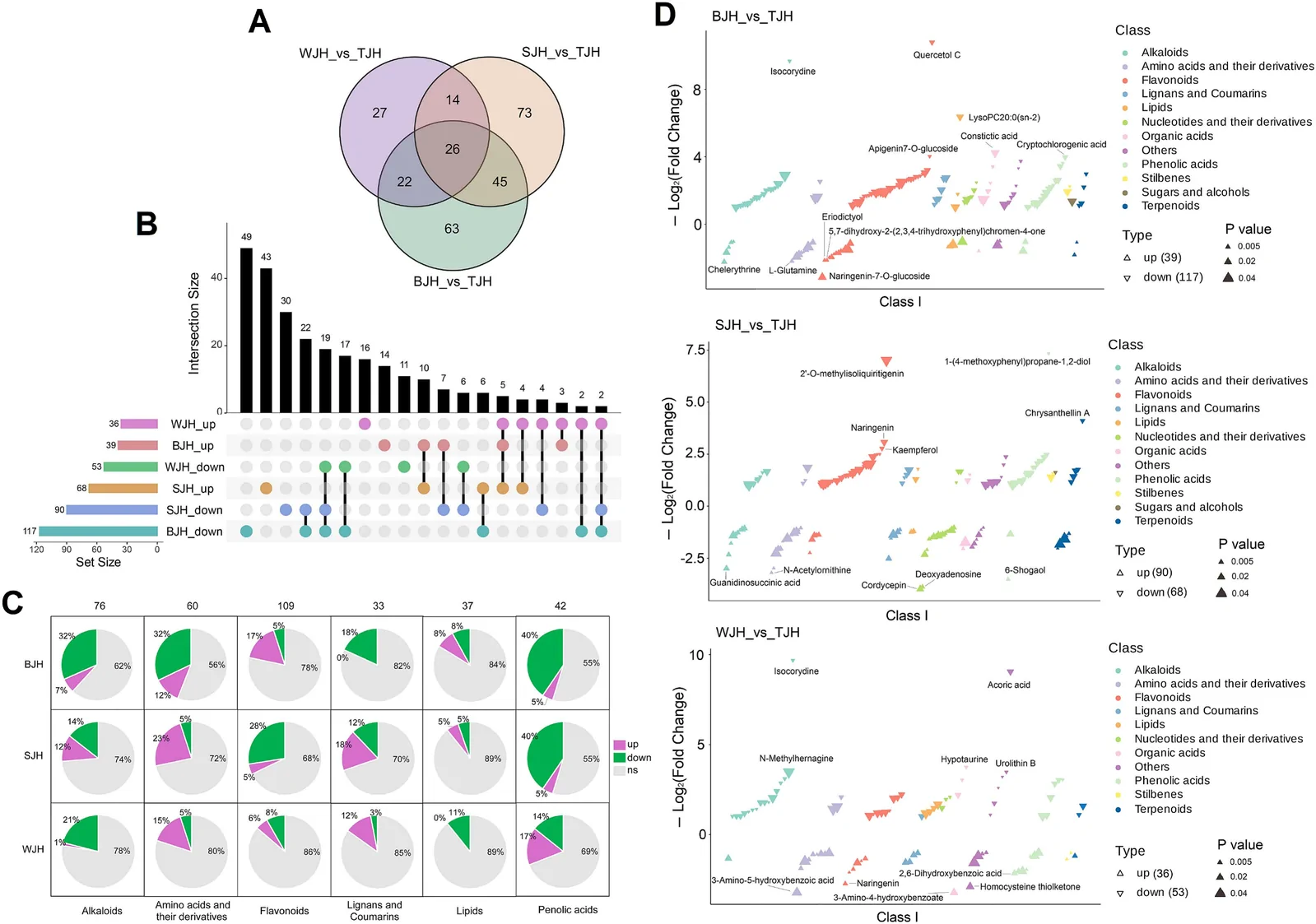
Metabolomic analysis of four Juhong tea samples. (A) Advanced Venn diagrams and (B) UpSet plots of differentially regulated metabolites among the three pairwise comparisons (BJH_vs_TJH, WJH_vs_TJH, and SJH_vs_TJH) (*p* < 0.05 and VIP > 1). (C) Advanced pie and (D) Volcano plots of secondary metabolites for the comparisons BJH_vs_TJH, WJH_vs_TJH, and SJH_vs_TJH. Gray dots represent metabolites showing no significant difference (ns, *p* > 0.05 or VIP < 1). The top 5 upregulated and downregulated secondary metabolites with the greatest fold changes were indicated. TJH, traditional Juhong; BJH, single bud Juhong; WJH, wild Juhong; SJH, shaded Juhong. The full data set is available in Supporting Information, Table S5.

### 3.4 Common and key nonvolatile metabolites analysis

We analyzed the top 50 most abundant metabolites in the quality control samples, comprising 13 flavonoids, 13 amino acids and their derivatives, 6 alkaloids, 4 terpenoids, 4 nucleotides and their derivatives, 3 organic acids, 3 lipids, 3 others, and 1 phenolic acid. These metabolites represented the common chemical constituents that may contribute to the taste characteristics of Juhong teas (Table S6). Among them, theanine, 2-aminoisobutyrate, and 1-[4-hydroxy-3-(2-hydroxyethyl)phenyl]ethan-1-one were the most enriched, exhibiting umami, sweet, and vanilla flavors, respectively. Among the 13 flavonoids, tangeritin and hesperidin were characteristic metabolites of citrus peels, contributing to a faint bitter taste. Kaempferol derivatives and delphinidin are commonly found in tea and have been reported to contribute to astringency. The coexistence of these metabolites is not merely a simple mixture; rather, their interactions collectively shape a unique flavor profile and functional network. Based on OPLS-DA results with VIP >1, 193 differential metabolites that were significantly altered among the four Juhong teas were identified (Table S7, Fig. 5), and a heatmap was constructed to visualize their abundance patterns. Metabolic profiling revealed distinct chemical signatures that may explain the sensory divergence among the four Juhong teas.

**Fig. 5:**
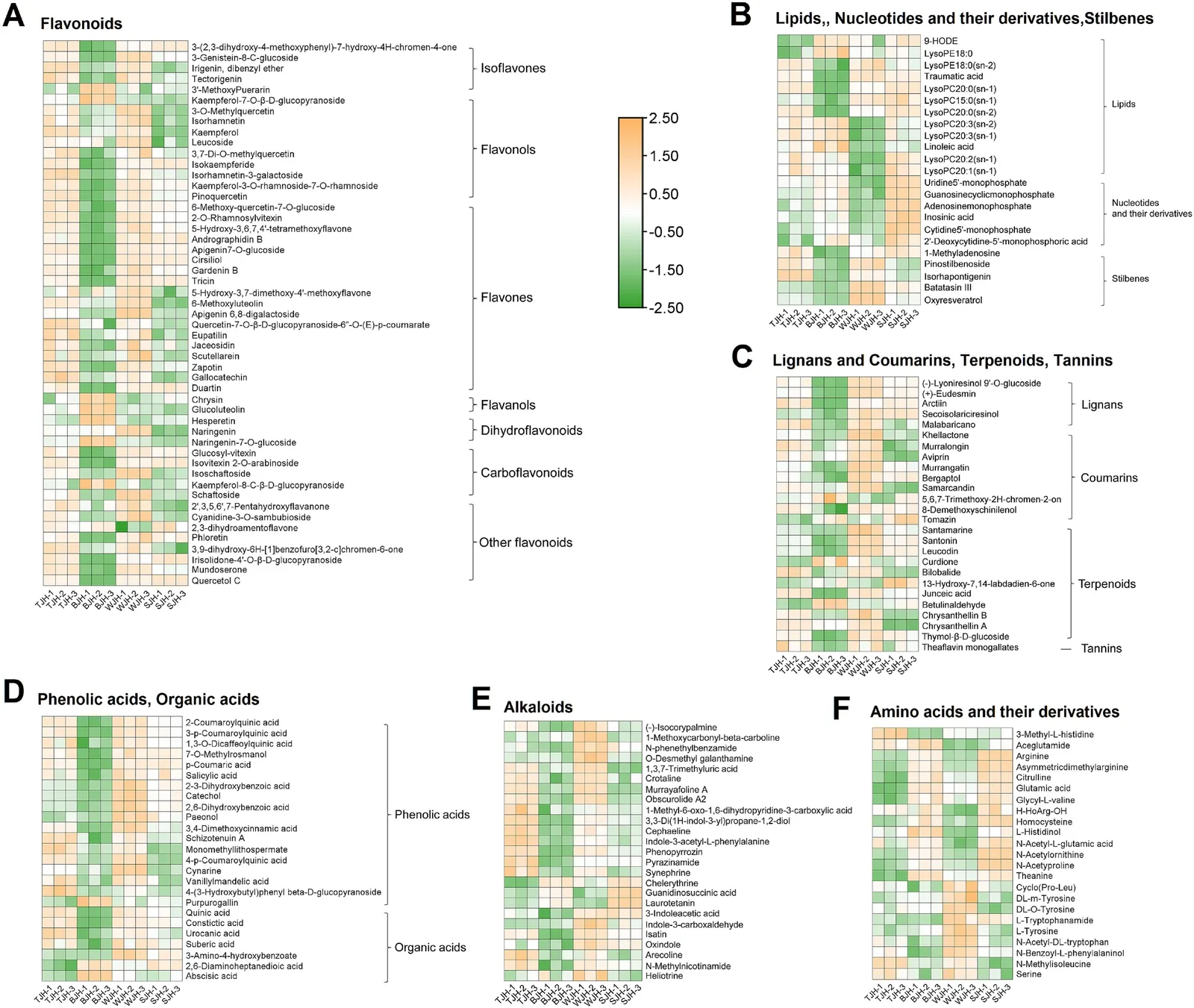
Heatmap of significant alteration metabolites in (A) flavonoids, (B) lipids, nucleotides and their derivatives, stilbenes, (C) lignans and coumarins, terpenoid, and tannins, (D) phenolic acids and organic acids, (E) alkaloids, and (F) amino acids and their derivatives. TJH, traditional Juhong; BJH, single bud Juhong; WJH, wild Juhong; SJH, shaded Juhong. The color scale is shared globally across all panels, representing the *Z*-score normalized values. Green indicates lower abundance, while orange indicates higher abundance. (For interpretation of the references to color in this figure legend, the reader is referred to the web version of this article.)

#### 3.4.1 Flavonoids

Flavonoids are critical determinants of not only the sensory quality of Juhong teas but also their coloration and potential pharmacological activities (Zhang et al., 2019). In this study, 51 differential flavonoids were identified and visualized across all samples (Fig. 5A). The largest number of highly accumulated flavonoids was observed in WJH among the four Juhong teas. These flavonoids, particularly polymethoxyflavones and flavone glycosides, often impart a characteristic bitterness and contribute to a complex flavor profile (Drewnowski & Gomez-Carneros, 2000; Nogata et al., 2006). In tea plants, flavonols predominantly occur as glycosides, which contribute to the astringency and mouthfeel of the tea infusion (De Jesus et al., 2023; Silva et al., 2025). TJH exhibited higher levels of specific flavonols, such as kaempferol, isorhamnetin, and 3-*O*-methylquercetin. Interestingly, although BJH had lower overall flavonoid levels, specific flavanones, like hesperetin and naringenin-7-O-glucoside, were abundant, suggesting their unique contribution to the quality BJH. SJH had relatively higher levels of isoflavone derivatives, such as 3-genistein-8-C-glucoside and irisolidone-4’-O-β-D-glucopyranoside. These findings suggest that shading may specifically promote the accumulation of these flavonoids, there by differentiating the metabolic profile from those of TJH and WJH.

#### 3.4.2 Lipids, nucleotides, and their derivatives

Fig. 5B showed that SJH was the most abundant in specific lipids (LysoPC 20:0, 15:0) and nucleotides (inosinic acid, AMP, cGMP), whereas WJH lacked these metabolites but was richer in stilbenes such as batatasin III and oxyresveratrol. While lipids such as LysoPCs were reported to impart a silky, smooth mouthfeel and a lingering coating sensation (Li et al., 2021), nucleotides such as IMP are potent flavor enhancers that synergize with theanine to enhance tea umami (Kaneko et al., 2006; Narukawa et al., 2014). In contrast, BJH was distinguished by lower levels of stilbenes, such as isorhapontigenin and pinostilbenoside.

#### 3.4.3 Lignans and coumarins, terpenoid, and tannins

WJH exhibited a significantly higher abundance of lignans and coumarins than other samples, whereas BJH showed the lowest levels of these compounds. Terpenoids, especially triterpenoid saponins (Chrysanthellin A/B), were more abundant in WJH but less abundant in SJH. Triterpenoids contribute to the characteristic bitterness of various foods and beverages, such as cucumber (Horie et al., 2007), kuding tea (Wüpper et al., 2020), and bitter melon (Deng et al., 2022). Among the tannins, theaflavin monogallates were the most abundant in TJH, followed by WJH. Theaflavins are critical determinants of the brightness, briskness, and richness of black tea infusions (Zhang et al., 2019). The superior richness score (0.27) of TJH in the electronic may be partly attributed to the its high theaflavin content, potentially through interactions with other components such as soluble proteins, contributing to a robust and mellow sensory experience.

#### 3.4.4 Phenolic acids and organic acids

Phenolic acids, primarily hydroxycinnamic acids and their derivatives, are key bioactive components that significantly influence tea flavor (Ge et al., 2021; Lai et al., 2022; Oliveras-López et al., 2016). For instance, *trans*-4-O-*p*-coumaroylquinic acid is a low-threshold contributor to the astringency of black tea (Wen et al., 2022). WJH showed a markedly higher accumulation of phenolic acids, with 2,3-dihydroxybenzoic acid and 2-coumaroylquinic acid contents approximately 7.8- and 5.9-fold higher than those of BJH. Organic acids are not only identified as the principal drivers of sourness in black tea (Yu et al., 2022) but also contribute to its mellow flavor (Song et al., 2020). For instance, quinic acid contributes to both tea sourness and astringency (Zhuang et al., 2020) while inducing cream and sediment formation through its interaction with Ca^2+^ (Xu et al., 2013). TJH and WJH exhibit the highest organic acid levels, particularly in quinic acid, approximately 2.6-fold higher than those in BJH.

#### 3.4.5 Alkaloids

Alkaloids contribute to bitterness in tea; their diminished levels during the withering stage of black tea processing help mitigate perceived bitterness and astringency (Dai et al., 2025; Xie et al., 2026). A total of 25 key differential alkaloids and related metabolites were identified in four Juhong teas (Table S6, Fig. 5E). Most alkaloids were found in higher concentrations in WJH and TJH, while their levels are lower in BJH. For instance, Obscurolide A2, one of the most prominent alkaloid components among the 25 alkaloids, showed markedly lower levels in BJH and SJH with respective fold changes of 0.33 and 0.41. Indole acts as a pivotal odor-active compound in Japanese matcha and has been proposed as a quality indicator for Sencha (Jumtee et al., 2011; Tan et al., 2019), both processed from fresh leaves after shading. Notably, compared with TJH, indole-3-carboxaldehyde and oxindole were significantly enriched in SJH and WJH, with a fold change of 1.43, 1.36 in SJH and 2.51, 1.62 in WJH, whereas these alkaloids were generally lower in BJH, with a fold change of 0.62 and 0.72, respectively. These quantitative variations in alkaloid profiles provide a chemical explanation for the distinct bitterness and flavor characteristics observed across the different Juhong tea samples.

#### 3.4.6 Amino acids and their derivatives

Amino acids and their derivatives are important precursors and flavor-active constituents that shape the sensory attributes and bioactive properties of tea. Among them, theanine is the most abundant free amino acid, contributing to umami and sweet notes accompanied by mild astringency and a caramel-like fragrance. Additionally, it suppresses bitterness and astringency while amplifying perceived sweetness (Yu & Yang, 2020). A total of 23 differentially abundant amino acids and their derivatives were identified among the four Juhong teas. With the exception of 3-methyl-L-histidine, aceglutamide, *N*-methylisoleucine, and serine, most compounds, were present at low levels in in TJH. BJH and SJH displayed similar metabolic profiles, characterized by notable enrichment of theanine and glutamic acid (umami), homocysteine (sweetness), and arginine (bitterness) (Geng et al., 2026), along with low levels of bitter aromatic amino acids such as tyrosine and tryptophan derivatives (Li et al., 2024). In contrast, WJH exhibited the opposite pattern.

### 3.5 Correlation analysis between sensory flavor attributes and taste-active metabolites

The final taste perception of Juhong tea is governed not only by the abundance of its chemical constituents but, more significantly, by the dynamic synergy and antagonism between its flavor-active compounds. These interactions shape the intensity of key sensory attributes, including umami, sweetness, bitterness, and astringency. While umami and sweetness are the characteristic of premium Juhong tea preferred by the consumers, bitterness and astringency often regarded as negative quality indicators. Therefore, a comprehensive investigation of the key taste-active compounds and their interactions is essential for understanding the overall palatability of Juhong tea. Correlation analysis was performed a to investigate the relationships between seven sensory attributes and the 34 taste compounds that were absolutely quantified. (Fig. 6). Fig. 6A showed that umami scores were strongly positively correlated with Glu (*r* = 0.76, *p* < 0.01), Thea (*r* = 0.72, *p* < 0.05), and TAAs (*r* = 0.69, *p* < 0.05). It has been reported that the interaction between L-theanine and other free amino acids is crucial for reducing the ratio of bitterness to sweetness, thereby improving the overall fresh and sweet quality of the infusion (Zhang et al., 2019). In contrast, ester-type catechins such as EGCG, CG, and ECG showed negative correlations with umami scores.

**Fig. 6:**
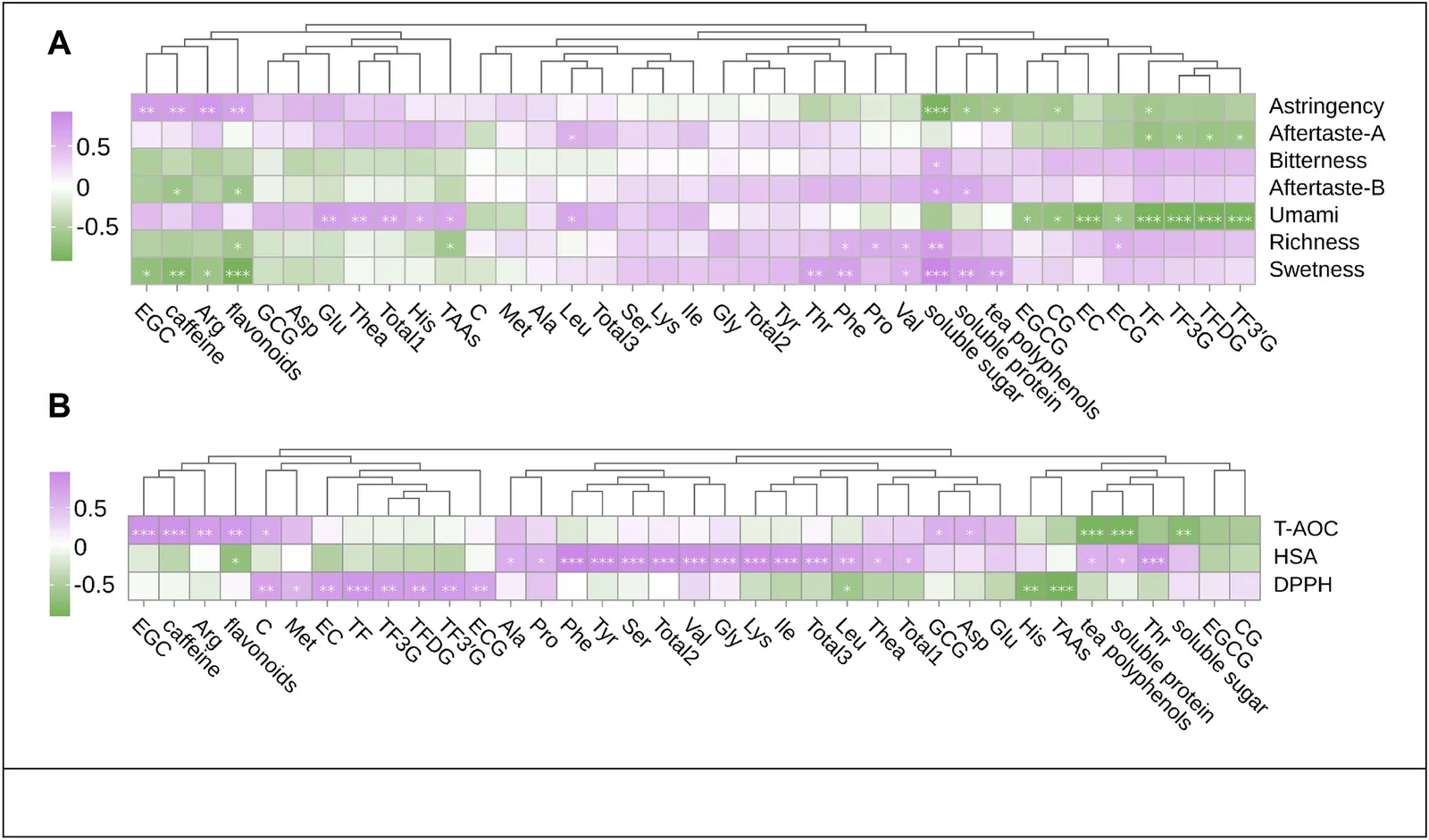
Heatmap of correlation analysis between chemical constituents and sensory attributes (A) and antioxidant activities (B) of Juhong teas. Asterisks indicate statistical significance based on correlation analysis: * *p* < 0.05, ** *p* < 0.01, and *** *p* < 0.001.

Astringency was positively correlated with EGC (*r* = 0.77, *p* < 0.01), caffeine (*r* = 0.76, *p* < 0.01), and flavonoids (*r* = 0.72, *p* < 0.05). Furthermore, theaflavins showed consistently positive correlations with richness and sweetness. These results confirm that catechins, caffeine, and flavonoids are the primary chemical drivers of astringency, as evidenced by their strong positive correlations. However, astringency perception is influenced by other factors. The presence of TFs and flavonol glycosides appears to modulate the sensory environment, potentially lowering the threshold for astringency perception through complex tongue-surface interactions (Scharbert et al., 2004). This highlights the complexity of tea flavor chemistry, where components like TFs serve both as flavor enhancers and modulators, lowering the perceptual threshold for astringent compounds.

Importantly, there was a strong positive correlation between soluble sugars and sweetness (*r* = 0.94, *p* < 0.001), as well as richness (*r* = 0.77, *p* < 0.01), and significant negative correlations with astringency (*r* = −0.66, *p* < 0.001) and umami (*r* = −0.56, *p* < 0.01). This suggests that soluble sugars are direct contributors to taste intensity and texture while simultaneously acting as sensory modulators that mask astringency. This phenomenon likely arises from both biochemical interference with polyphenol-salivary protein binding (Kaneko et al., 2006) and cognitive competition between basic tastes.

### 3.6 Evaluation of in vitro antioxidant activity

Tea-derived bioactive constituents, particularly polyphenols, exhibit potent free radical scavenging capacity (Tang et al., 2019). Citrus peel flavonoids exhibit antioxidant activity through multiple mechanisms, including free radical neutralization, suppression of lipid peroxidation, and metal ion chelation (Chen et al., 2017). In this study, the four Juhong tea samples showed significant differences in radical-scavenging activity abilities of various Juhong tea samples when tested against the same radical, and each sample demonstrated unique levels of effectiveness. Similarly, individual samples exhibited distinct antioxidant activity depending on the radical tested. SJH demonstrated the highest total antioxidant capacity (T-AOC), whereas BJH exhibited superior hydroxyl radical-scavenging activity (HSA) but the lowest DPPH radical-scavenging capacity, suggesting a possible preference for hydroxyl radicals (·OH). TJH displayed the highest DPPH radical-scavenging activity, while WJH had weaker antioxidant effects, particularly in HSA (Fig. S4). These differences likely result from varying phytochemical compositions among the four Juhong teas.

A correlation heatmap (Fig. 6B) was created to illustrate the relationship between antioxidant capacities and the 34 functional components of Juhong teas. The radical-scavenging efficacy of polyphenolic compounds is primarily governed by the number and positionof phenolic hydroxyl groups within their molecular structures(Yan et al., 2020). T-AOC exhibited pronounced positive correlations with EGC (*r* = 0.88, *p* < 0.001), flavonoids (*r* = 0.82, *p* < 0.01), C (*r* = 0.70, *p* < 0.05), and GCG (*r* = 0.64, *p* < 0.05). The antioxidant role of amino acids is largely determined by their side-chain features. Groups containing sulfur or aromatic rings help scavenge reactive oxygen species, whereas others facilitate chelation of metal ions, thereby providing a secondary defense against oxidation (Kim et al., 2020; Yang et al., 2023). Significant positive correlations with HSA were observed for Phe (*r* = 0.97), Tyr (*r* = 0.95), and Ser (*r* = 0.90) (all *p* < 0.001), and T-AOC was strongly correlated with Arg (*r* = 0.80, *p* < 0.01) and Asp (*r* = 0.65, *p* < 0.05). DPPH radical scavenging activity was strongly correlated with TF (*r* = 0.86, *p* < 0.001), TF3G (*r* = 0.81, *p* < 0.01), and TFDG (*r* = 0.81, *p* < 0.01). This indicates that the benzotropolone ring in TF is a highly active site for hydrogen atom transfer. TFs reportedly exhibit potent DPPH scavenging activity, often exceeding that of certain simple catechins, due to their specific oxidative coupling structures (Leung et al., 2001). Thus, the antioxidant capacity of Juhong tea may stem from the synergistic action of polyphenols, amino acids, and theaflavins.

## 4 Conclusion

In this study, we systematically integrated sensory evaluation, electronic-tongue profiling, biochemical assays, and widely targeted metabolomics to delineate the impact of four diverse black tea raw materials (TJH, BJH, WJH, and SJH) on the flavor chemistry and antioxidant properties of Juhong tea. Our findings revealed that raw material characteristics markedly dictate the flavor balance and functional profiles of the composite tea. BJH exhibited the highest overall sensory acceptance, driven by substantial levels of free amino acids and balanced polyphenolic constituents, whereas TJH presented superior sweetness and mouthfeel richness associated with high soluble sugar and theaflavin concentrations. A total of 193 discriminatory nonvolatile metabolites, prominently flavonoids, phenolic acids, and amino acid derivatives, were identified as key drivers of inter-sample flavor divergence. Furthermore, distinct in vitro radical-scavenging capacities demonstrated that the antioxidant potential of Juhong tea is collectively driven by the synergy among theaflavins, polyphenols, and specific free amino acids.

Nonetheless, this work focused predominantly on nonvolatile taste-active metabolites, the contribution of volatile aroma profiles and the intricate volatile–nonvolatile perceptual interactions during brewing remain to be fully characterized. Additionally, the in vivo bioavailability and physiological mechanisms underlying Juhong tea's antioxidant benefits warrant further validation. In conclusion, our study elucidates the chemical foundation of taste formation in Juhong tea and provides practical theoretical guidelines for raw material selection, processing optimization, and standardized quality control in citrus-based composite tea manufacturing.

## CRediT authorship contribution statement

**Anhui Gui:** Writing – original draft, Visualization, Validation, Methodology, Investigation, Formal analysis, Data curation, Conceptualization. **Fei Ye:** Visualization, Validation, Methodology, Investigation, Formal analysis, Data curation. **Lin Feng:** Visualization, Validation, Software, Investigation. **Shengpeng Wang:** Methodology, Formal analysis. **Panpan Liu:** Validation, Investigation, Data curation. **Jinbo Liang:** Resources, Investigation. **Xueping Wang:** Investigation, Data curation. **Jinjin Xue:** Validation, Methodology, Formal analysis. **Jun Xiang:** Resources, Investigation. **Fangjun Su:** Resources, Investigation. **Jing Teng:** Methodology, Formal analysis. **Jing Huang:** Investigation, Data curation. **Pengcheng Zheng:** Writing – review & editing, Project administration, Investigation. **Shiwei Gao:** Writing – review & editing, Project administration, Conceptualization.

## Declaration of competing interest

The authors declare the following financial interests/personal relationships which may be considered as potential competing interests: Reports a relationship with that includes:. Has patent pending to. If there are other authors, they declare that they have no known competing financial interests or personal relationships that could have appeared to influence the work reported in this paper.

## Data Availability

Data will be made available on request.
